# Retention in care in aging adults with a dual diagnosis of HIV infection and type 2 diabetes mellitus: a longitudinal retrospective cross-sectional study

**DOI:** 10.1186/s12981-020-00286-z

**Published:** 2020-05-29

**Authors:** Julie Ann Zuniga, Alexandra A. García, Junse Lee, Jungmin Park

**Affiliations:** 1 School of Nursing, The University of Texas at Austin, Austin, TX USA; 2 Division of Community Engagement and Health Equity, Department of Population Health, Dell Medical School, The University of Texas at Austin, Austin, TX USA; 3 Electrical and Computer Engineering, The University of Texas at Austin, Austin, TX USA; 4 School of Nursing, Hanyang University, 222 Wangsimni-ro, Sageun-dong, Seongdong-gu, Seoul, South Korea

**Keywords:** CD4 count, Type 2 diabetes mellitus, HIV, Retention in care, Comorbidity

## Abstract

**Background:**

This study aimed to investigate the measures of retention in care (RIC) in persons living with HIV (PLWH) and type 2 diabetes mellitus (T2DM) by age group (younger vs. older adults).

**Methods:**

This was a longitudinal retrospective cross-sectional study that used secondary data from the Center for AIDS Research Network of Integrated Clinical Systems (CNICS). We examined RIC in 798 adult PLWH + T2DM who visited a CNICS clinic at least once in 2015. Six measures of RIC were examined: missed visits [measured as a continuous variable (total number of missed visits) and dichotomous variable (0 = never missed, 1 = missed)], visit adherence, 6-month visit gap, 4-month visit constancy, and the Health and Resources Services Administration HIV/AIDS Bureau’s RIC measure. We calculated Spearman correlation coefficients and conducted logistic regression and multi-group path analysis.

**Results:**

Most RIC measures were significantly correlated (p < 0.05) with one another; only 4-month visit constancy was not correlated with other measures. Except for the number of missed visits in older adult PLWH + T2DM, we found no significant relationships between RIC measures and CD4 cell count using logistic regression. However, multi-group path analysis demonstrated significant positive relationships between most RIC measures and CD4 cell count in both age groups. In younger adults living with HIV (YALWH) + T2DM, HbA1c level, but not CD4 count, was significantly associated with most RIC measures.

**Conclusions:**

RIC is related to disease control (CD4 cell count and HbA1c level) in PLWH + T2DM and notably, HbA1c level was only significantly affected in YALWH + T2DM. A future study is needed to find more accurate reasons for the fact that only HbA1c level had significant relationships in YALWH + T2DM. The findings from this study provide guidance in measuring RIC in PLWH who have comorbidities.

## Background

Presently, life expectancy for > 1.2 million persons living with HIV (PLWH) is comparable to that for the general population [1, 2], in part because of the development of effective antiretroviral therapy (ART) [3]. However, as PLWH age, more than half develop at least one comorbidity [4]. In older adults (aged ≥ 50 years) living with HIV (OALWH), type 2 diabetes mellitus (T2DM) is one of the most common comorbidities [5–8]. Approximately 10–15% of PLWH have T2DM, a prevalence rate slightly higher than that in persons without HIV [6, 9, 10]. Because the prevalence of T2DM increases with age and the population of older adults with T2DM is expected to increase in the future [11], the same trend is also likely to be reflected in the PLWH population.

In 2014, OALWH accounted for 17% of PLWH, and the proportion is growing [12–17]. Because OALWH have more comorbidities than younger adults with HIV (YALWH, aged 19–49 years), OALWH encounter unique barriers to HIV management [18]. Approximately 30% of OALWH have at least two chronic conditions [19], and controlling both HIV infection and T2DM is the key to survival, complication prevention, and maintenance of quality of life. To achieve long-term control of HIV infection, PLWH must visit their healthcare providers at regular intervals, an important predictor of health called retention in care (RIC). RIC is important in successful HIV management because it enables the monitoring of combination ART, evaluation of medication toxicity, and identification of HIV treatment failure [20]. Additionally, RIC has been associated with the ultimate goal of HIV care, i.e., undetectable HIV viral load [21]. Undetectable viral load greatly decreases the risk of HIV transmission [22].

It is recommended that PLWH visit their healthcare provider every 3–6 months for blood tests so that their condition can be monitored. Once the virus is stable and undetectable, PLWH may decrease the number of annual visits [23]. Approximately 51% of PLWH are retained in care after diagnosis [24]. However, even though PLWH retained in care can expect improved survival rates [25] and RIC has been associated with positive HIV outcomes, it is unknown whether RIC is also associated with positive outcomes in those with comorbidities, such as T2DM.

RIC can be measured using several different methods including missed appointments, number of appointments, and number of appointments within a certain time frame [26]. However, not all RIC measures accurately reflect health benefits and risks, depending on patients’ disease progression, ART initiation, and other medical conditions that may need close monitoring, such as T2DM. This study aimed to investigate the relationships of RIC measures in PLWH + T2DM in two age groups (older vs. younger adults) and explore the relationships between RIC and health outcomes relevant to HIV infection and T2DM.

## Methods

### Research design

This was a longitudinal retrospective cross-sectional study that used secondary analysis of data from the Center for AIDS Research (CFAR) Network of Integrated Clinic Systems (CNICS), which includes medical records of PLWH from 1995. The longitudinal retrospective cross-sectional study design was based on the study by Mugavero et al. [26] which set the “gold standard” of measurements in PLWH regardless of comorbidities. This study was exempt from review by the Institutional Review Board of the University of Texas at Austin because the data were already de-identified.

### Measures

The CNICS database contains medical records of > 32,000 patients from eight CFAR clinics throughout the USA, including 798 PLWH + T2DM aged > 18 years who received ART from CNICS clinics for at least 6 months and had at least one primary care appointment at a CNICS clinic in 2015. Diabetes diagnosis was verified by HbA1c levels > 6.5% or use of medications for T2DM. The dataset excluded patients with type 1 diabetes because our focus was on PLWH + T2DM. Data included age (≥ 50 years old, < 50 years old), sex, race/ethnicity, number of clinic visits in 2015, and CD4 count, which was analyzed at CNICS clinics. Three groups were created based on the CD4 count: ≥ 500 cells/μL, 200–499 cells/μL, and < 200 cells/μL [27]. Six RIC measures were used: missed clinic visits (count, dichotomous), visit adherence, 4-month visit constancy, 6-month visit gap, and the US Health Resources and Services Administration HIV/AIDS Bureau (HRSA HAB) measure.

- Missed visits were unkept appointments that were not cancelled by the patient or clinic staff. Missed visits were captured continuously (summed for the total number of missed visits) and dichotomously (0 = kept the visit, 1 = missed the visit). More missed visits indicate worse RIC.
- Visit adherence is a continuously measured variable of the proportion of appointments kept versus that of the appointments scheduled. More kept visits show better RIC.
- Four-month visit constancy is the sum of 4-month intervals with ≥ one kept visit (range, 0–3).
- Six-month visit gap is the number of episodes with ≥ 189 days between kept visits [0 = not retained, 1 = retained (≥ one visit in 6 months)].
- HRSA HAB measure (90-day gap) is attendance at visits ≥ 90 days apart [0 = not retained, 1 = retained (two kept visits separated by ≥ 90 days in a 12-month period)] [21].

For health outcome variables, we obtained two outcome variables for PLWH + T2DM representing HIV and T2DM control: CD4 count at the last visit and most recent HbA1c level, respectively. A CD4 count < 200 cells/µL indicates the last stage of HIV infection. In AIDS, a CD4 count > 500 cells/µL represents a normal value, and a count between 200 cells/µL and 500 cells/µL indicates HIV treatment with sustained good health status [27]. HbA1c level reflects the level of glycemia for 3 months [28], an indicator of T2DM management [29]. T2DM control in PLWH is represented by HbA1c levels < 6.5% [30, 31].

### Statistical analysis

Descriptive statistics using means, standard deviations, ranges of scores, frequencies, and percentages were applied to analyze demographic characteristics [age, sex, race/ethnicity, missed visits (count, dichotomous), attendance, visit adherence, 4-month visit constancy, 6-month visit gap, HRSA HAB measure, HbA1c level, and CD4 count]. Spearman’s correlation coefficients were used to evaluate associations among six RIC measures [missed visits (count, dichotomous), visit adherence, 4-month visit constancy, 6-month visit gap, HRSA HAB measure], health outcomes (CD4 count, HbA1c level), and age groups (≥ 50 years, < 50 years). Logistic regression was used to evaluate RIC measures and age groups as predictors of HIV status determined by CD4 count. Logistic regression was also used to predict the symptom burden in PLWH + T2DM (as measured by the HIV symptom index) using age group and RIC measure [using the HRSA HAB measure (not retained vs. retained 90-day gap)]. Finally, to investigate the relationships between RIC measure and disease control (CD4 count, HbA1c level) by age group, we conducted multi-group path analysis. In the path analysis, missed visits (count), visit attendance (count, actual visit clinic times), visit adherence, and 4-month visit constancy for RIC were considered independent variables, and the CD4 count and HbA1c level were considered dependent variables. The Chi square, normed fit index (NFI), Tucker–Lewis Index (TLI), comparative fit index (CFI), and root mean square error of approximation (RMSEA) were used to estimate model fit. Statistical significance in all tests was set at α = 0.05. The statistical analyses were conducted using IBM SPSS version 23.0 [32] and AMOS version 23.0 [33].

## Results

All patients in this study (*N* = 798) were PLWH + T2DM. They were predominantly male (77%), aged 55 years, and fairly racially and ethnically diverse (41% White non-Hispanic, 40% African American non-Hispanic, 18% Hispanic). They had fairly well-controlled conditions (mean HbA1c level = 7.6%; mean CD4 count = 718) and were retained in care (missed visits days average: 0.29 day) with an average of three visits in 2015 (Table 1).

**Table 1:** PLWH + T2DM characteristics

Characteristics	PLWH + T2DM All age groups (*N* = 798)	OALWH + T2DM (*n* = 564)	YALWH + T2DM (*n* = 234)	Pearson Chi Square or Fisher Exact ^a^(OALWH + T2DM, YALWH + T2DM)
Age (years) mean ± SD	55.01 ± 8.96	59.35 ± 7.22 (70.7%)	44.56 ± 5.10 (29.3%)	
Gender				0.01**
Male	618 (77.4%)	451 (80%)	167 (71.4%)	
Female	180 (22.6%)	113 (20%)	67 (28.6%)	
Race/ethnicity	*N *= 794	*N* = 560		0.001***
White—non-Hispanic	326 (41.1%)	253 (45.2%)	73 (31.2%)	
White—Hispanic	98 (12.3%)	62 (11.1%)	36 (15.4%)	
African American—non-Hispanic	320 (40.3%)	214 (38.2%)	106 (45.3%)	
African American—Hispanic	3 (0.4%)	0 (0%)	3 (1.3%)	
Hispanic	18 (2.3%)	12 (2.1%)	6 (2.6%)	
Others	29 (3.7%)	19 (3.4%)	10 (4.3%)	
Missed visits (count)	Range: 0–12	Range: 0–5	Range: 0–12	0.23
Mean ± SD	0.29 ± 0.90	0.24 ± 0.66	0.41 ± 1.30	
Zero	667 (83.6%)	476 (84.4%)	191 (81.6%)	
One	84 (10.5%)	61 (10.8%)	23 (9.8%)	
Two	27 (3.4%)	16 (2.8%)	11 (4.7%)	
≥ Three	20 (2.4%)	11 (2%)	9 (3.7%)	
Missed visits (dichotomous)				0.35
No missed visits	667 (83.6%)	476 (84.4%)	191 (81.6%)	
At least one missed visit	1 (16.4%)	88 (15.6%)	43 (18.4%)	
Visit attendance	Range: 0–26 days	Range: 0–26 days	Range: 0–26 days	0.01**
Mean ± SD	3.07 ± 2.517	3.05 ± 2.417	3.12 ± 2.747	
Visit adherence				0.06
Mean ± SD	0.904 ± 0.24	0.910 ± 0.23	0.890 ± 0.25	
0–24%	33 (4.1%)	23 (4.1%)	10 (4.3%)	
25–49%	15 (1.8%)	12 (2.1%)	3 (1.3%)	
50–74%	62 (7.9%)	34 (6%)	28 (11.6%)	
75–99%	47 (5.9%)	37 (6.6%)	10 (4.2%)	
100%	641 (80.3%)	458 (81.2%)	183 (78.2%)	
4-month visit constancy (intervals with ≥ 1 kept visit)	*n* = 772	*n* = 546	*n* = 226	0.67
Zero	7 (0.9%)	5 (0.9%)	2 (0.9%)	
One	207 (26.8%)	143 (26.2%)	64 (28.3%)	
Two	334 (43.3%)	244 (44.7%)	90 (39.8%)	
Three	224 (29%)	154 (28.2%)	70 (31%)	
6-month gap (≥ 189 days between sequential kept visits)	*n* = 606	*n* = 425	*n* = 181	0.19
Retained	570 (94.1%)	396 (93.2%)	174 (96.1%)	
Not retained	36 (5.9%)	29 (6.8%)	7 (3.9%)	
HRSA HAB measure (2 kept visits > 90 days apart)	*n* = 606	*n* = 425	*n* = 181	0.39
Retained	418 (69%)	298 (70.1%)	120 (66.3%)	0.07
Not retained	188 (31%)	127 (29.9%)	61 (33.7%)	
HbA1c level	*n* = 179	*n* = 179	*n* = 179	0.07
Mean ± SD	7.565 ± 1.93	7.529 ± 1.90	7.69 ± 2.03	
≤ 6.5%	64 (35.8%)	50 (36%)	14 (35%)	
> 6.5%	115 (64.2%)	89 (64%)	26 (65%)	
CD4 count	*n* = 194	*n* = 151	*n* = 43	0.61
Mean ± SD	717.84 ± 407.42	696.44 ± 387.06	792.98 ± 469.35	
≥ 500 cells/μL	132 (68%)	102 (67.5%)	30 (69.8%)	
200–499 cells/MlμL	49 (25.3%)	40 (26.5%)	9 (20.9%)	
< 200 cells/μL	13 (6.7%)	9 (6%)	4 (9.3%)	

Some variables have different sample sizes

*HRSA HAB measure* the US Health Resources and Services Administration HIV/AIDS Bureau (HRSA HAB) measure, *PLWH + T2DM* persons living with HIV and diabetes, all ages, *OALWH + T2DM* older adults with HIV and T2DM, *YALWH + T2DM* younger adults with HIV and T2DM

* *p *< 0.05, ** *p *< 0.01, ***p < 0.00

^a^Pearson Chi Square or Fisher Exact between OALWH + T2DM and YALWH + T2DM

Older adults (> 50 years, *n* = 564) had a mean age of 59 years, and younger adults (*n* = 234) were approximately 15 years younger, with a mean age of 45 years. The two groups were statistically similar in almost all RIC measures and CD4 count and HbA1c level (*p *> 0.05). However, there were significant differences in sex, race/ethnicity, and attendance (*p* < 0.05) between the older and younger adults. There were more older men (80%) than younger men (71%; *p* = 0.01) in the sample and more African American non-Hispanic patients among younger adults (45.3%; *p* = 0.001) than among older adults (40.3%; Table 1).

### RIC

To evaluate the relationships among the six RIC measures in PLWH + T2DM, we conducted Spearman’s correlations (Table 2). Dichotomously measured missed visits were highly positively correlated with continuously measured missed visits (*r* = 0.996, *p* < 0.01). Both measures of missed visits (count, dichotomous) were highly negatively correlated with visit adherence (*r* = − 0.854, *p* < 0.01). However, 4-month visit constancy and 6-month visit gap had a minimally negative correlation (*r* = − 0.105, *p* < 0.01), but neither demonstrated significant correlations with missed visits (either count or dichotomous) or visit adherence. The HRSA HAB measure was minimally positively correlated with visit adherence (*r* = 0.130, *p *< 0.01) and 6-month visit gap (*r* = 0.169, *p *< 0.01) and negatively correlated with missed visits (both count and dichotomous) (*r* = − 0.141 and − 0.140, respectively, *p* <* 0*.01). The HRSA HAB measure had a high positive correlation with 4-month visit constancy (*r* = 0.93, *p* < 0.05).

**Table 2:** Spearman’s correlation coefficients of RIC measures for PLWH + T2DM, all age groups

	Missed visits (count)	Missed visits (dichotomous)	Visit adherence	4-month visit constancy	6-month gap	HRSA HAB measure
Missed visits (count, range: 0–12)	1					
Missed visits (dichotomous)	0.996**	1				
Visit adherence (continuous, range: 0–1)	− 0.854**	− 0.854**	1			
4-month visit constancy (categorical, range: 0–3)	0.005	0	0.33	1		
6-month gap (dichotomous)	0.02	0.024	− 0.029	− 0.105**	1	
HRSA HAB measure (dichotomous)	− 0.141**	− 0.140**	0.130**	0.93*	0.169**	1

Some variables have different sample sizes

*HRSA HAB measure* the US Health Resources and Services Administration HIV/AIDS Bureau (HRSA HAB) measure, *PLWH *+ *T2DM* persons living with HIV and diabetes, all ages, *OALWH + T2DM* older adults with HIV and T2DM, *YALWH + T2DM* younger adults with HIV and T2DM

* *p* < 0.05, ** *p* < 0.01

#### OALWH + T2DM

Spearman’s correlations for all RIC measures in older adults showed almost the same pattern as those in the whole sample of PLWH (Table 3). As in the whole sample, missed visits (count and dichotomous) had a highly positively correlation and were highly negatively correlated with visit adherence. Similarly, 4-month visit constancy and 6-month visit gap had a significant association but were not significantly correlated with missed visits (count, dichotomous) or visit adherence. Unlike that in PLWH + T2DM, the HRSA HAB measure in OALWH + T2DM was not correlated with 4-month visit constancy.

**Table 3:** Spearman’s correlation coefficients of RIC measures for OALWH + T2DM

	Missed visits (count)	Missed visits (dichotomous)	Visit adherence	4-month visit constancy	6-month gap	HRSA HAB measure
Missed visits (count, range: 0–5)	1					
Missed visits (dichotomous)	0.997**	1				
Visit adherence (continuous, range: 0–1.0)	− 0.854**	− 0.855**	1			
4-month visit constancy (categorical, range: 0–3)	0.022	0.023	0.11	1		
6-month gap (dichotomous)	− 0.009	− 0.003	− 0.002	− 0.126**	1	
HRSA HAB measure (dichotomous)	− 0.143**	− 0.143**	0.130**	0.05	0.177**	1

*HRSA HAB measure*, the US Health Resources and Services Administration HIV/AIDS Bureau (HRSA HAB) measure, *OALWH *+ *T2DM* older adults with HIV and T2DM

** *p* < 0.01

#### YALWH + T2DM

The pattern of correlations for YALWH + T2DM differed from those for PLWH + T2DM and OALWH + T2DM (Table 4). Most notably, the HRSA HAB measure had a low positive correlation with 4-month visit constancy (*r* = 0.182, *p* < 0.05) but was not correlated with other RIC measures. Moreover, unlike those in OALWH + T2DM and the entire sample, 4-month constancy in YALWH + T2DM was not related to the 6-month visit gap.

**Table 4:** Spearman’s correlation coefficients of RIC measures for YALWH + T2DM

	Missed visits (count)	Missed visits (dichotomous)	Visit adherence	4-month visit constancy	6-month gap	HRSA HAB measure
Missed visits (count, range: 0–12)	1					
Missed visits (dichotomous)	0.994**	1				
Visit adherence (continuous, range: 0–1.0)	− 0.852**	− 0.853**	1			
4-month visit constancy (categorical, range: 0–3)	− 0.027	− 0.050	0.074	1		
6-month gap (dichotomous)	0.11	0.111	− 0.123	− 0.55	1	
HRSA HAB measure (dichotomous)	− 0.133	− 0.131	0.125	0.182*	0.143	1

*HRSA HAB measure*, the US Health Resources and Services Administration HIV/AIDS Bureau (HRSA HAB) measure; *YALWH *+ *T2DM*, younger adults with HIV and T2DM

* *p* < 0.05, ** *p *< 0.01

#### Health outcomes

We investigated the relationship between RIC and CD4 count (Table 5). In OALWH + T2DM (*n* = 105), the only significant RIC measure that significantly predicted CD4 count was the number of missed visits [Exp(B) = 11.277, 95% confidence interval (CI) (1.499, 84.843), *p* < 0.05], indicating that having more missed visits predicted an 11-times greater chance of having a CD4 count < 500 cells/µL (Hosmer–Lemeshow Chi square, *p* < 0.05). However, no RIC measures predicted CD4 count in YALWH + T2DM (*n* = 34).

**Table 5:** Associations between retention in care measures and CD4 cell count (< 500 cells/µL) in PLWH + T2DM by age groups

Age group	Variables^a^	B	*SE*	Wald	*df*	Sig.	Exp(B)	95% CI	
								Lower	Upper
OALWH + T2DM (*n* = 105)	Missed visits (count)	2.423	1.03	5.537	1	0.019*	11.277	1.499	84.843
	Missed visits (dichotomous)	2.069	1.716	1.454	1	0.228	7.918	0.274	228.713
	Visit adherence	5.582	5.733	0.948	1	0.33	265.714	0.004	20153270.43
	4-month visit constancy	0.685	0.566	1.464	1	0.226	1.983	0.654	6.014
	6-month gap	0.407	0.864	0.222	1	0.638	1.502	0.276	8.173
	HRSA HAB measure (retained)	0.082	0.618	0.018	1	0.894	1.086	0.323	3.648
	Constant	− 9.479	5.378	3.107	1	0.078	0		
	Total Chi square (6) = 10.421, *p *> 0.05								
	Hosmer–Lemeshow Chi square (3) = 0.89, *p *> 0.05								
YALWH + T2DM (*n* = 34)	Missed visits (count)	10.804	16,337.328	0	1	0.999	49,219.776	0	
	Missed visits (dichotomous)	30.158	62,616.205	0	1	1	1.25186E + 13	0	
	Visit adherence	4.645	235,894.115	0	1	1	104.095	0	
	4-month visit constancy	0.185	1.645	0.013	1	0.911	1.203	0.048	30.251
	HRSA HAB measure (retained)	− 0.849	1.465	0.336	1	0.562	0.428	0.024	7.56
	Constant	− 35.14	180,003.341	0	1	1	0		
	Chi square (6) = 7.07, *p *> 0.05								
	Hosmer–Lemeshow Chi square (4) = 2.29, *p *> 0.05								

*HRSA HAB measure* the US Health Resources and Services Administration HIV/AIDS Bureau (HRSA HAB) measure, *PLWH *+ *T2DM* persons living with HIV and diabetes, all ages, *OALWH *+ *T2DM* older adults with HIV and T2DM, *YALWH *+ *T2DM* younger adults with HIV and T2DM

* *p* < 0.05

^a^Step 1^a^. variables: missed visits (count), missed visits (dichotomous), visit adherence, 4-month visit constancy, 6-month gap, HRSA HAB measure; Step 1^b^. variables: missed visits (dichotomous), visit adherence, 4-month visit constancy, HRSA HAB measure

#### RIC and disease control paths

**Fig. 1:**
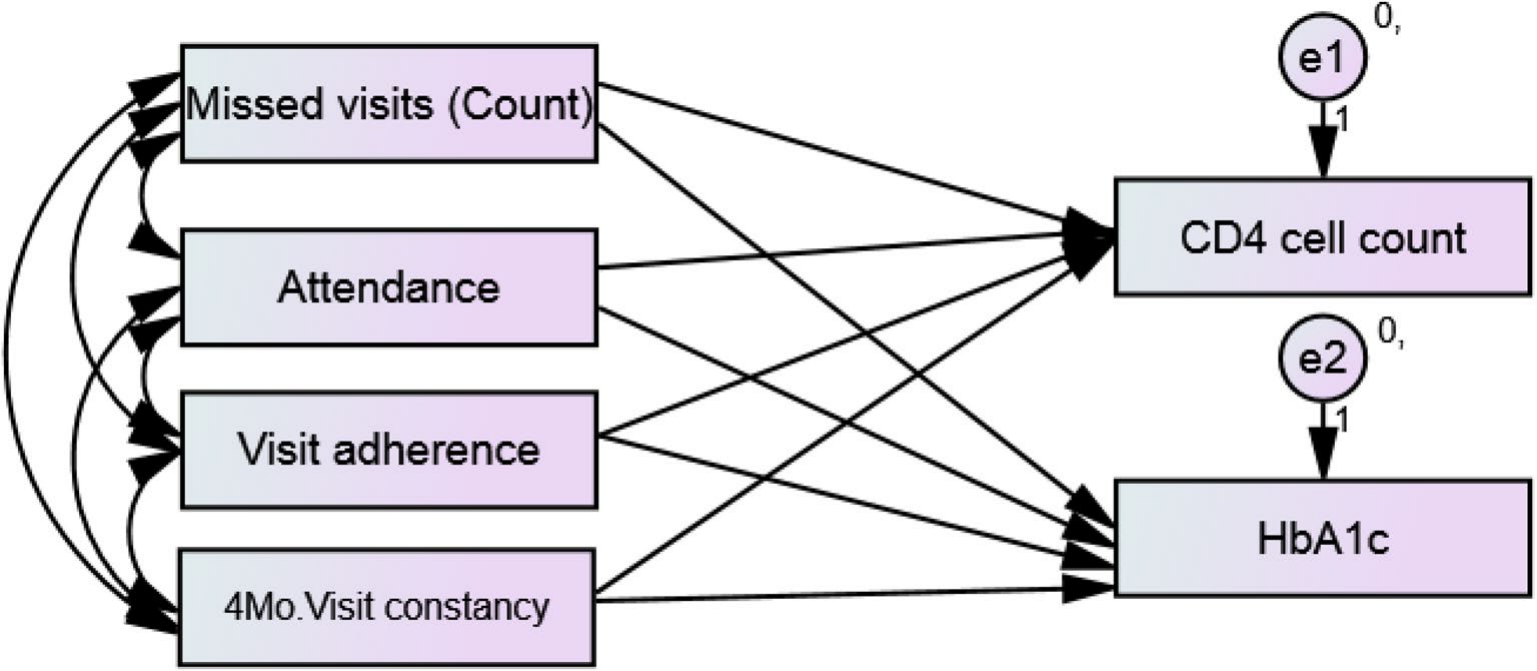
Hypothesized path model in PLWH + T2DM. *e1* error 1, *e2* error 2, *4Mo.* 4 month

**Fig. 2:**
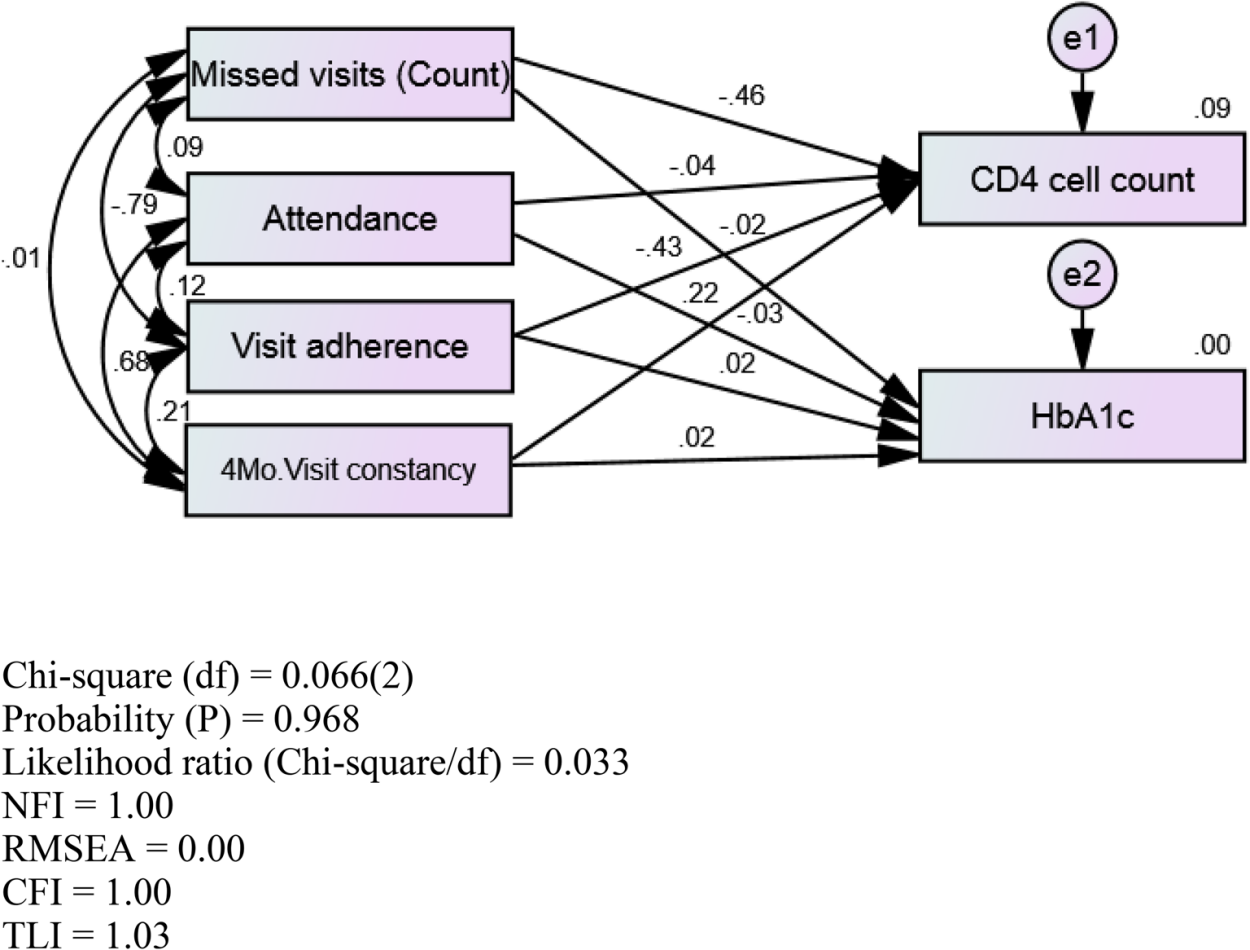
Hypothesized model with path coefficients in OALWH + T2DM. *CFI* comparative fit index, *e1* error 1, *e2* error 2, *4Mo.* 4 month, *NFI* normed fit index, *RMSEA* root mean square error of approximation, *TLI* Tucker–Lewis Index

**Fig. 3:**
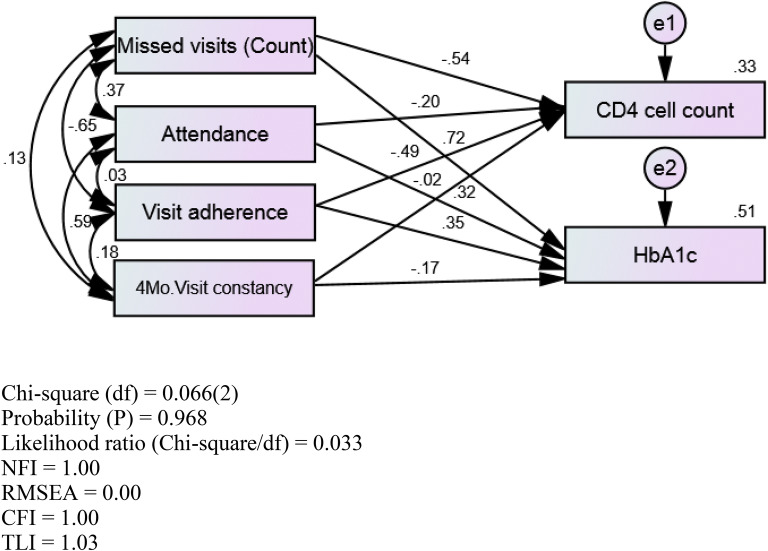
Hypothesized model with path coefficients in YALWH + T2DM. *CFI* comparative fit index, *e1* error 1, *e2* error 2, *4Mo*. 4 month, *NFI* normed fit index, *RMSEA* root mean square error of approximation, *TLI* Tucker–Lewis Index

Path analysis was conducted to assess relationships among RIC measures [missed visit (count), attendance, visit adherence, 4-month visit constancy, and disease control (CD4 cell count, HbA1c level)] in OALWH + T2DM and YALWH + T2DM (Table 6). Figure 1 presents the hypothesized path model for PLWH + T2DM, and Figs. 2 and 3 show the hypothesized models with path coefficients for OALWH + T2DM and YALWH + T2DM, respectively. Path analysis for OALWH + T2DM showed three significant hypothesized paths [missed visits (count), visit adherence, 4-month visit constancy] in CD4 count (*p* < 0.05) (Table 6). The model fit was good. Missed visits (count), visit adherence, and 4-month visit constancy were significantly related to CD4 count (*p* < 0.05). However, there were no significant relationships among missed visits (count), attendance, visit adherence, 4-month visit constancy, and HbA1c level ($\chi$^2^(2) = 0.066, *p* = 0.968, NFI = 1.0, RMSEA = 0.00, CFI = 1.00, TLI = 1.03) (*n* = 546). Visit adherence was significantly related to missed visits (count) and 4-month visit constancy (*p* < 0.05). Path analysis showed some differences between YALWH + T2DM and OALWH + T2DM. In YALWH + T2DM ($\chi$^2^[2] = 0.066, *p* = 0.968, NFI = 1.0, RMSEA = 0.00, CFI = 1.00, TLI = 1.03, *n* = 226; Table 7), missed visits (count) and visit adherence were significantly related to CD4 cell count (*p* < 0.05). Missed visits (count), attendance, and visit adherence were significantly related to HbA1c level (*p* < 0.05). Moreover, missed visits (count) were significantly related to visit attendance and adherence (*p* < 0.05). The model fit was good.

**Table 6:** Standardized path coefficients by age groups

Model	Path	Path coefficient	*p*
OALWH + T2DM	Standardized effects on CD4 cell count		
	Missed visits (count)	–0.46	0.001
	Attendance	–0.04	0.73
	Visit adherence	–0.43	0.002
	4-month visit constancy	0.22	0.040
	*Standardized effects on HbA1c level*		
	Missed visits (count)	–0.02	0.89
	Attendance	–0.03	0.78
	Visit adherence	0.02	0.90
	4-month visit constancy	0.024	0.84
YALWH + T2DM	Standardized effects on CD4 cell count		
	Missed visits (count)	–0.54	0.001
	Attendance	–0.20	0.26
	Visit adherence	–0.50	0.01
	4-month visit constancy	–0.02	0.91
	Standardized effects on HbA1c level		
	Missed visits (count)	0.72	0.001
	Attendance	0.32	0.04
	Visit adherence	0.35	0.03
	4-month visit constancy	–0.17	0.23

*PLWH *+ *T2DM* persons living with HIV and diabetes, all ages, *OALWH *+ *T2DM* older adults with HIV and T2DM, *YALWH *+ *T2DM* younger adults with HIV and T2DM

**Table 7:** Model fit comparisons by age group

Age group	χ^2^	*df*	*P*	NFI	RMSEA	CFI	TLI
OALWH + T2DM	0.066	2	0.968	1.0	0.00	1.00	1.03
YALWH + T2DM	0.066	2	0.968	1.0	0.00	1.00	1.03

For all models, p < 0.05

*CFI* comparative fit index, *NFI* normed fit index, *TLI* Tucker–Lewis Index, *RMSEA* root mean square error of approximation, *PLWH + T2DM* persons living with HIV and diabetes, all ages, *OALWH + T2DM* older adults with HIV and T2DM, *YALWH + T2DM* younger adults with HIV and T2DM

## Discussion

The patients in this study had few missed visits, and the large majority (84%) had not missed any visits in the year. Moreover, 72% had attended visits in two or three 4-month periods in a year, and 94% did not have a gap of 6 months between appointments. More than two-thirds had attended two visits in the year with at least 90-day intervals. This is higher than the national average reported by the Centers for Disease Control and Prevention, which reported that 57.7% of their sample (N = 358,151) was retained in care [32]. Almost two-thirds were in poor T2DM control and more than two-thirds of the sample had good control of HIV. Thus, the sample was relatively healthy. This could be attributed to the CNICS clinic. The clinics in this consortium offer a variety of services and opportunities to be a part of research studies. Being connected to a larger HIV community may be protective.

In this study, we examined the differences in RIC measures among PLWH in two age groups. The two different age groups had largely similar characteristics. The difference in ethnicity between the age groups may be accounted for by the shorter life expectancy of African American PLWH [33]. OALWH + T2DM had significantly lower visit attendance than YALWH + T2DM (*p* = 0.01). This result could possibly be due to insufficient access of OALWH + T2DM to clinic services, perhaps because older adults are more likely to have inadequate social support than younger adults (e.g., they may live alone or lack health insurance) [18]. Thus, some OALWH simply might not visit the clinic.

We also assessed the usefulness of six RIC measures for the sample and by age group. Spearman’s correlations revealed differences in measures in PLWH + T2DM. Four-month visit constancy was not correlated with other RIC measures. This might be because 4-month visit constancy, unlike other measures, was assessed in 12 months and divided into 4-month segments. PLWH + T2DM might not have visited the clinic in each 4-month segment because they are not frequently scheduled appointments if their HIV status is under control. However, Mugavero et al. [26] who did not consider PLWH and additional comorbidities, found that all RIC measures, including the 4-month visit constancy, had significant correlations in PLWH. Perhaps, 4-month visit constancy may not be an appropriate measure for PLWH with comorbidities.

Logistic regression showed that only missed visits (count) significantly predicted CD4 count in OALWH + T2DM; other measures were not significantly associated with CD4 count. We expected stronger correlations, as reported by Mugavero et al. [26]. However, Mugavero’s sample population was younger with more female and African American individuals. The major difference was that all patients in our sample were diagnosed with T2DM, which may change RIC if more appointments are necessary to manage each condition. This disconnect between missed appointments and higher CD4 count, may mean that patients with track records of strong immune systems did not feel it necessary to make as many appointments, or were able to get refills on their medication without an in-person visit. Changes in the increased availability of tele-health visits may see improvements in the amount of missed visits. Further research is needed in this area.

Lastly, this study performed path analysis for factors affecting the CD4 count and HbA1c levels in PLWH + T2DM divided into two age groups. Missed visits affected CD4 counts regardless of age, suggesting that RIC is important in HIV disease control (*p* < 0.05). Most RIC measures were significantly correlated with HbA1c level in YALWH + T2DM but not in OALWH + T2DM (*p* < 0.05).

These results are partially consistent with those of Zuniga et al. [34] who found that OALWH have better control of T2DM than YALWH. However, one main area of T2DM control in PLWH, i.e., RIC in older adults was not significantly correlated with the HbA1c level. This may be because T2DM is a disease that emphasizes self-management—daily glucose monitoring, dietary management [35], and medication adherence [36]—and perhaps because OALWH + T2DM are more motivated or able to manage T2DM than YALWH + T2DM [36].

An important contribution of this study is the investigation of the interrelationships of RIC measures. We found that RIC can positively affect comorbidities and HIV infection, at least in the older population.

### Limitations

Although the CNICS dataset represents a large, nationally representative sample of PLWH, this study has several limitations. The sample population comprised only those who visited the eight clinics in the CNICS network, which might limit generalizability to other populations. The study did not include those who might have died in calendar year 2015; thus, the results could be biased toward healthier patients. CD4 was selected over viral load because of there was less missing data with CD4. Since there were missing data on CD4 count and HbA1c levels that could have led to bias in the statistical analysis. Future studies should aim to include more complete samples of CD4 count and HbA1c levels in PLWH + T2DM.

## Conclusions

We conducted a comparative analysis on RIC in PLWH + T2DM who are part of a large national cohort study in one calendar year. RIC measures predicted CD4 count regardless of age. Nonetheless, the HbA1c level was significantly affected in YALWH + T2DM. This is the first study to consider PLWH + T2DM in terms of age groups. Even though our study contained a limited sample of CD4 count and HbA1c level data, our results will guide research on the relationships between RIC and CD4 counts and HbA1c levels in PLWH + T2DM in the future.

## Data Availability

The data that support the findings of this study are available from the Center for AIDS Research (CFAR) Network of Integrated Clinical Systems (CNICS), but restrictions apply to the availability of these data, which were used under license for the current study, and so are not publicly available. Data are however available from the authors upon reasonable request and with permission of CNICS.

## Abbreviations

ART: Antiretroviral therapy
CFAR: Center for AIDS Research
CFI: Comparative fit index
CNICS: Center for AIDS Research Network of Integrated Clinical Systems
NFI: Normed fit index
OALWH: Older adults living with HIV
PLWH: Persons living with HIV
RIC: Retention in care
RMSEA: Root mean square error of approximation
TLI: Tucker–Lewis Index
YALWH: Younger adults living with HIV
